# Fat-water separated myocardial T_1 _mapping with IDEAL-T_1 _saturation recovery gradient echo imaging

**DOI:** 10.1186/1532-429X-16-S1-P65

**Published:** 2014-01-16

**Authors:** Joseph J Pagano, Kelvin Chow, Ray Yang, Richard B Thompson

**Affiliations:** 1 Biomedical Engineering, University of Alberta, Edmonton, Alberta, Canada

## Background

Myocardial T_1 _mapping is emerging as powerful tool for tissue characterization, however the presence of intramyocardial or epicardial fat can contaminate T_1 _values through partial voluming, or preclude analysis, particularly in areas of infarct or thin walled myocardium, such as the right ventricle. We propose and evaluate a new combined fat-water separated saturation-recovery imaging sequence (IDEAL-T_1_) for water-separated T_1 _mapping.

## Methods

The IDEAL-T_1 _approach combines a gated, segmented multi-echo gradient recalled echo readout for fat-water separation, based on the "iterative decomposition of water and fat with echo asymmetry and least squares estimation" (IDEAL) method[1], with saturation recovery T_1 _mapping[2-4]. Images at 4 saturation recovery (TS) times were acquired at a basal slice in diastole over 2 breathholds; one for a non-saturation prepared image, with >4 seconds of recovery between segments, and another for 3 images with incremental TS times. Typical parameters: (Siemens Sonata, 1.5T) TE 2.06, 4.43, 6.8 ms, TR 8.59, flip angle 20°, TS 302-701 ms, FOV 360 × 259 mm, acquisition matrix 256 × 129, phase resolution 70%, 6/8 partial Fourier, 27 views per segments (4 shots per image). Data from water-separated images was scaled by the non-saturated image and fit to a 1-parameter mono-exponential curve, using a Bloch equations simulation look-up table approach to correct for readout-effects on apparent saturation efficiency. In phantom experiments, with a physiologic range of T_1 _and T_2 _values (14 phantoms), IDEAL-T_1 _was validated against an inversion spin-echo sequence. In-vivo evaluation of myocardial T_1 _was completed in 6 healthy individuals and compared to a single-shot saturation recovery sequence (SASHA)[2] in the left ventricle.

## Results

**Figure 1:**
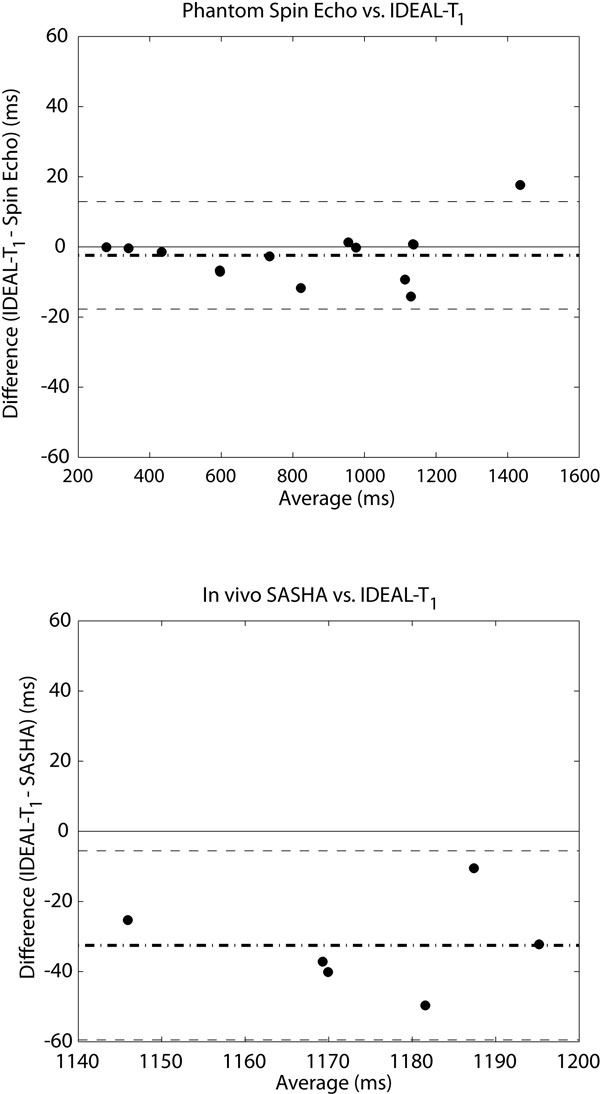
**Bland-Altman analysis for phantom (top) and in vivo (bottom) experiments**.

**Figure 2:**
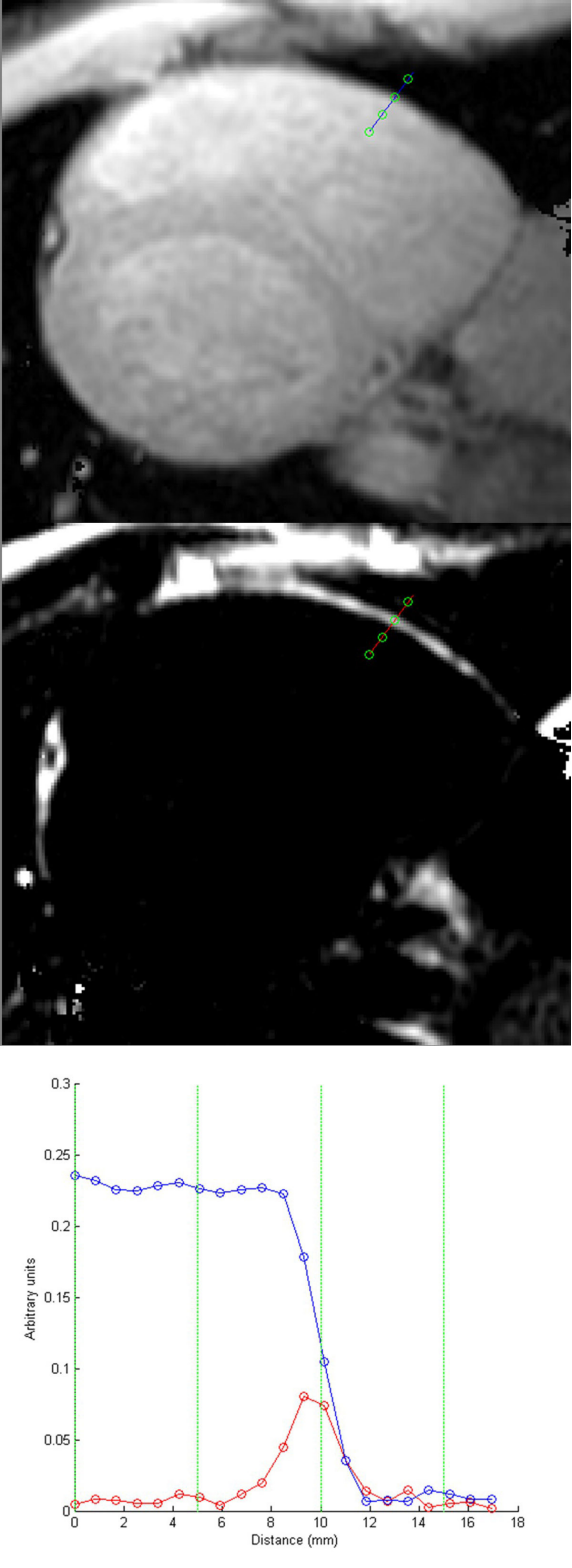
**IDEAL-T_1 _images showing typical water separated image (top), fat separated image (middle), and signal profile (bottom) across the right ventricle free wall**. A small rim of fat is not otherwise visible unless noted on fat separated image.

Simulations reveal negligible dependence on T_1_, T_2_, and off-resonance (up to 250 Hz), but dependence on B1 errors and saturation efficiency. Phantom experiments show excellent correlation with spin-echo values (R2 0.9996, p < 0.0001) with a mean underestimation of 2.4 ms (Figure 1) and a standard deviation of the difference of 7.4 ms. In vivo evaluation shows a larger underestimation, with a mean difference of -32.5 ms (Figure 1) and a standard deviation of the difference of 12.3 ms. Sample fat and water separated images are shown in Figure 2, where a thin rim of RV fat is revealed on the fat image, and a fat and water profile through the wall illustrates the large region of fat and water overlap.

## Conclusions

IDEAL-T_1 _provides the benefit of fat-water separation with quantitative myocardial T_1_-mapping with a small underestimation in T_1_. Areas of thin myocardium, including the right ventricle, may benefit from resolving zones of partial voluming with fat by having water only images for analysis.

## Funding

The authors acknowledge financial support from CIHR, AIHS, WCHRI.
